# Wood Veneer Defect Detection Based on Multiscale DETR with Position Encoder Net

**DOI:** 10.3390/s23104837

**Published:** 2023-05-17

**Authors:** Yilin Ge, Dapeng Jiang, Liping Sun

**Affiliations:** College of Computer and Control Engineering, Northeast Forestry University, No. 26 Hexing Road, Harbin 150040, China

**Keywords:** wood veneer, defect detection, convolutional neural networks, transformer

## Abstract

Wood is one of the main building materials. However, defects on veneers result in substantial waste of wood resources. Traditional veneer defect detection relies on manual experience or photoelectric-based methods, which are either subjective and inefficient or need substantial investment. Computer vision-based object detection methods have been used in many realistic areas. This paper proposes a new deep learning defect detection pipeline. First, an image collection device is constructed and a total of more than 16,380 defect images are collected coupled with a mixed data augmentation method. Then, a detection pipeline is designed based on DEtection TRansformer (DETR). The original DETR needs position encoding functions to be designed and is ineffective for small object detection. To solve these problems, a position encoding net is designed with multiscale feature maps. The loss function is also redefined for much more stable training. The results from the defect dataset show that using a light feature mapping network, the proposed method is much faster with similar accuracy. Using a complex feature mapping network, the proposed method is much more accurate with similar speed.

## 1. Introduction

Wood has many advantages such as being malleable, environmentally friendly, renewable, etc. Therefore, it has been used in many areas [1]. Since the dawn of human history, wood has been a main building material. Even today, in some scenic areas of China, there are still some wooden temples and palaces, which were built hundreds or even thousands of years ago. Furthermore, wood is the main raw material for paper making. In some remote areas, wood is still used as the primary energy source. Nowadays, wood is still one of the most important industrial raw materials [2]. With the explosive growth of the world’s population, the demand for wood is increasing substantially. However, trees and forest resources play an irreplaceable role in the global environment. Excessive felling of trees will bring irreversible negative consequences to forests, and apparently, to the global environment. Therefore, how to improve the utilization rate of wood has become a research hotspot in academia in recent years [3,4].

Veneer is the raw product of logs. It is mainly used to produce plywood, joinery board, formwork, veneer panels and other artificial wood boards, etc. [5]. Due to the intrinsic characteristics and the influence of the growing environment, there are always some live knots, dead knots and so on, in logs. These defects have a great influence on the appearance and quality of the veneer. In some extreme cases, defects can affect normal use and even cause serious consequences [6]. Due to the existence of defects, many low-quality veneers are abandoned, resulting in substantial waste of wood resources. By detecting defects in advance, veneer can be graded according to the quality and workers can take measures to dredge and repair the defective areas. How to quickly and accurately detect the type and contour of veneer surface defects plays a vital role in improving the utilization rate and quality of veneer. Therefore, veneer defect detection has been an indispensable part of the whole veneer processing field and has drawn great attention from researchers [2,5].

Traditional detection methods can be classified into two categories, manual-based detection and photoelectric-based detection [7]. Manual-based detection relies on a large amount of labor, resulting in high labor costs. In addition, manual-based detection is not only inefficient, but also excessively dependent on people’s work experience, given that the detection results are very subjective. Different people often give different results for the same defect. The other drawback of manual-based detention is that the detection results can not be utilized directly in the automatic post-processing procedure. Photoelectric-based detection methods include the X-ray method, infrared-based method, laser-based methods and so on. Although photoelectric-based detection methods are much more efficient compared to the manual-based method, the investment in the early stage is much higher, and the detection results are not intuitive. Furthermore, these methods cannot accurately classify the defect categories.

Benefitting from the development of computer vision technology, especially the explosive development of deep learning, image processing-based object detection methods are becoming more and more mature. These methods are widely used in many areas, such as vehicle detection [8], ship detection [9], face recognition [10], agricultural pest monitoring [11], etc., as well as defect detection [5,6]. Compared to the manual-based and photoelectric-based methods, computer vision-based, more precisely, deep learning-based defect detection methods are much more efficient and accurate, and are easy employed [9,11]. Deep learning does not need features to be manually designed like traditional methods, and has greater abstract learning and generalization ability. Therefore, they have become mainstream approaches in academia and industry.

Convolutional neural networks (CNN) [12] is one of the most popular deep learning algorithms. Since 2012, all of the winning entries of ILSVRC [13] have been designed based on CNN, such as AlexNet [12], VGG [14], SENet [15], etc. The error rate of Top-5 and Top-1 of ImageNet is refreshed every year. The depth of the network has increased from 8 layers at the beginning to more than 1000 layers, and the width of the network is also increasing. At present, CNN is the dominant model in computer vision, and many lightweight models have been proposed, such as EfficientNet [16] and MobileNet [17], etc. On the other hand, Transformer [18], one deep learning model based on the attention mechanism, challenges CNN both in terms of speed and accuracy. Transformer-based models have achieved SOTA performances in many computer vision areas [19]. However, Transformer essentially learns the correlation information of sequences and cannot perceive the global image like CNN. Therefore, how to make use of CNN’s global perception ability and Transformer’s powerful logic correlation ability simultaneously needs to be studied further. Moreover, location coding in Transformer requires manual design, which is subjective and cannot obtain optimal results.

Based on the analyses above, in this paper, we propose a new defect detection pipeline. First, an image collection device is constructed and a total of more than 16,380 defect images are collected through a mixed data augmentation method, including live knots, dead knots and wormholes. Then, a detection pipeline is designed based on DETR [20]. A position encoding net is designed to replace the manually designed position encoding formula. In the backbone, multiscale feature maps are used to obtain fine-gained features. The loss function is also redefined for a much more stable training. The main contributions can be summarized as follows. (1) A panel defect detection dataset is produced containing three common defects, live knot, dead knot and wormhole. (2) A multiscale feature mapping network is designed to increase the detection performance for small defects. (3) The manually designed position encoding function is replaced by a self-learned network.

The rest of this paper is organized as follows. Section 2 briefly introduces some background. The dataset used in this paper and the related data augmentation methods are introduced in Section 3. Section 4 presents our proposed detection pipeline. Experimental results and analysis are given in Section 5. Section 6 concludes the paper and proposes future works.

## 2. Literature Review

### 2.1. Review of Classic Object Detection Methodologies

The problem definition of object detection is to determine where objects are located in a given image (object localization) and to which category each object belongs (object classification). Traditional object detection models can be divided into three stages: informative region selection, feature extraction and classification [21]. Informative region selection intends to produce candidate regions in which the considered objects may appear. Exhaustively searching all the windows can obtain a 100% recall rate, but the time cost is unacceptable. Therefore, a variety of papers offer methods for generating region candidates, such as objectness, selective search [22], etc. Feature extraction intends to extract features from the regions selected in the prior step. The representation ability of the extracted features has a substantial influence on the classification performance. Many classical feature extraction methods have been designed, such as scale-invariant feature transform (SIFT) [23], speeded-up robust features (SURF) [24], histogram of oriented gradient (HOG) [25], etc. In the classification stage, the extracted features are inputted into classifiers, such as SVM and K-nearest neighbors, to recognize the category of the related region. Traditional detection methods have been used in many realistic tasks in the pre-deep-learning period. However, manually designed features only contain low-level information, so the expression ability and description ability are always limited. Furthermore, these feature extraction methods are driven by expert knowledge and experience. These result in poor universality.

Taking advantage of the excellent deep representation ability of CNN, deep learning-based object detection methods have been proposed. Region-CNN (RCNN) [22], as a milestone of object detection in the deep learning era, is a two-stage architecture including region proposal and CNN-based feature selection. RCNN has a significant impact on the development of subsequent object detection methods. A family of RCNN-based detection methods have been proposed, such as fast-RCNN [26], faster-RCNN [27], mask-RCNN [28], etc.

You only look once (YOLO) [29] is another popular deep learning-based object detection method. Different from the two-stage methods, YOLO can be defined as a one-stage pipeline. Both the location and classification tasks are completed by a shared CNN model. Similarly, a series of YOLO-based methods have been proposed. Benefitting from the simple detection structure, the original YOLO is much faster than RCNN, coupled with low accuracy. However, the recently proposed YOLOv7 [30] has achieve high detection accuracy as well as faster detection speed. Other one-stage methods, such as SSD [31] and DSSD [32], also draw great attention.

The attention mechanism [18] is a set of methods used to model information in different locations. Existing methods based on the attention mechanism have been widely studied in tasks such as machine translation and speech recognition. Transformer is an outstanding method of natural language processing proposed by Google in 2017. Transformer combines the self-attention mechanism and does not use the recurrent neural network (RNN) sequence structure, enabling parallel training of models. It is able to capture global information and won the SOTA competition in natural language processing that year. Vision Transformer (ViT) [19] was the first work of Transformer in the task of image classification, and it obtained comparable performance compared to the SOTA results of CNN-based methods. This initiated Transformer research in the field of computer vision. In the same year, the first Transformer-based detection pipeline, namely DETR, was proposed. Compared to the CNN-based detection methods, DETR needs even fewer manually designed steps, such as non-maximum suppression, while maintaining the detection accuracy. Recently, a lot of DETR variants have been proposed, such as Deformable DETR [33], PnP-DETR [34], etc.

DETR shows comparable performance compared to the current state-of-the-art methods. However, the results for many large-scale datasets show that DETR fails to detect small objects [20]. This mainly because that the input feature map is downsampled many times. The detail information of the small objects is lost in the feature extraction procedure. Furthermore, inherited from Transformer, DETR needs positional encoding functions to be defined experientially, which is too subjective.

### 2.2. Research on Veneer Defect Detection

In the growth process of trees, affected by weather, diseases and pests, there will always be some natural defects, such as live knots, dead knots, etc. Furthermore, during mining and storing procedures, defects such as checking also will be caused. The task of veneer defect detection is to locate and recognize these defects for post-processing.

With regard to traditional defect detection, Danvind used computed tomography (CT) technology to carry out nondestructive testing on logs in order to obtain the information of structural characteristics and moisture, which provides guidance for rational utilization [35]. Sarigul detected important hardwood defects through the analysis of CT images of logs, considering defect-dependent post-processing methods based on mathematical morphology [36]. Bhandarkar took advantage of computer axial tomography (CAT) images to detect internal defects [37,38]. Qi constructed an approach of image edge detection [39]. López used infrared thermography for the exploration and detection of subsurface singularities and defects in wood [40].

With regard to deep learning-based methods, Shi constructed an integrated model to detect wood veneer defects [41]. Ma [42] designed an end-to-end veneer automatic grading system. Hu [43] identified wood defects using a combined deep neural network model. Fan used ResnetV2 to extract collected solid wood panel defect images for feature extraction [44]. Gao proposed a new TL-ResNet34 deep learning model to detect wood knot defects [45]. Yang proposed a method based on a single shot multibox detector algorithm to detect wood surface defects [46]. Xia [47] modified the original Faster-RCNN for veneer detection by improving the bilateral filtering algorithm to smooth the image texture background and a feature pyramid network with a shape-variable convolutional ResNet50 network as well as a region of interest align algorithm. Hu [48] proposed a defect detection network based on multi-scale feature extraction. He proposed a mixed, fully convolutional neural network (Mix-FCN) to detect the location of wood defects [49]. Ding used transfer learning to detect wood defects [50]. Yang [51] designed a detection system to identify four types of bark defects such as dead knots, slipknots, holes and cracks on the surface of the wood. The detection system can collect data in real time and quickly.

Traditional detection methods are not easily generalizable to other situations. For example, the investments required in the early stage are substantial, which limits the applications in realistic detection problems. Although the above deep learning-based methods obtained adequate performance, the detection speed is not adequate for real usage. This is mainly due to the complex feature abstraction neural networks. Moreover, nearly all the proposed detection networks are designed for particular problems. On the other hand, no DETR-based methods are exploited for veneer detection, and new methods are always necessary.

## 3. Data Preparation

**Figure 1:**
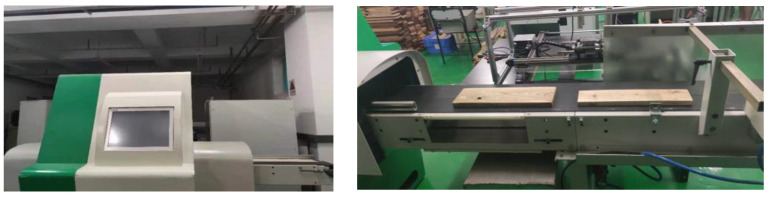
Image collection device.

Three kinds of defects are considered in this paper, i.e., live knot, dead knot and wormhole. An image collection device is constructed with a CCD camera, as shown in Figure 1. Veneers with defects are sent to the device at a constant speed. Uneven illumination may cause serious problems in image data acquisition. In order to achieve a high-quality dataset, an LED light array and uniform light panel are used to illuminate the surface of the veneer order to strictly control the light level. A total of 2730 images are collected, including 1000 images of live knot, 860 images of dead knot and 870 images of wormhole. The original images have 2048 × 2048 pixels. In order to accelerate the training and predicting speed of the proposed model, all the images are normalized to 512 × 512.

Data augmentation can ease overfitting caused by an insufficient original training dataset and increase the generalization ability of a learning model. Therefore, it has been used as a standard procedure before the training process. In this paper, a total of eight image augmentation methods are employed, including horizontal flipping, vertical flipping, cropping, affine transformation, one of three blur methods (Gaussian blur, average blur and median blur), add Gaussian noise, contrast normalization, piecewise affine and elastic transformation.

In contrast to the other papers that only use one augmentation method for one image, this paper undertakes the augmentation operation for one image using all of the methods mentioned above. Practically, for every image, only one of the two flipping methods will be employed, then all the other seven methods will be employed randomly in a 50% probability. Specifically, one original image will first be flipped horizontally or vertically, then the resulting image will be cropped 10% at a 50% probability, then a set of affine transformations will be conducted (scaling between 80%~120%, translation ±20%, rotation ±45°) at a 50% probability, then some of the following methods will be applied: blur, adding Gaussian noise, contrast normalization, piecewise affine and elastic transformation. After applying all the augmentation methods above, a new image will be obtained. The augmentation procedure is based on the Imgaug-0.4.0, a frequently used Python library.

**Figure 2:**
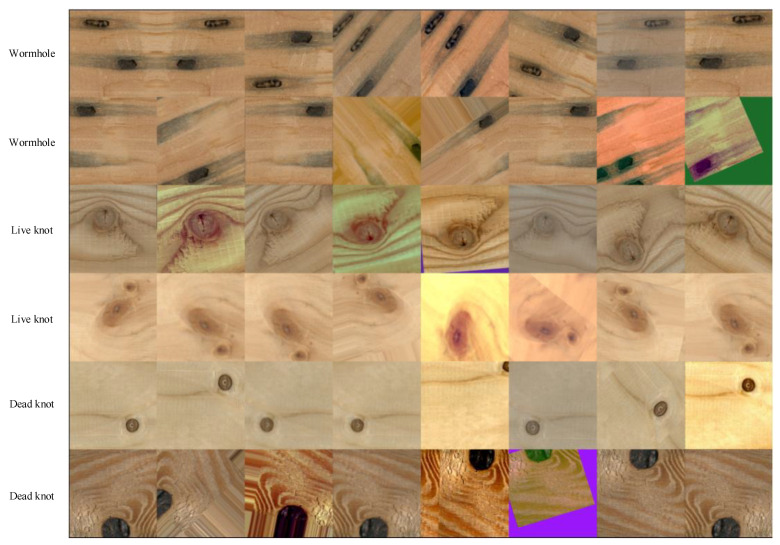
Image augmentation results. The first column presents the original images.

Figure 2 presents an example of the augmentation results. The first column is the original image, and the other columns are the augmentation results from the mixed operations mentioned above. For every image, augmentation is operated five times, which augments the original dataset to make it 6 times larger.

After all the augmentation has been completed, there are 16,380 images in total and all the images are annotated in a VOC format. The dataset is split into a training dataset, validation dataset and test dataset in proportions of 70%, 10% and 20%, respectively. The detailed dataset description is shown in Table 1.

**Figure 3:**
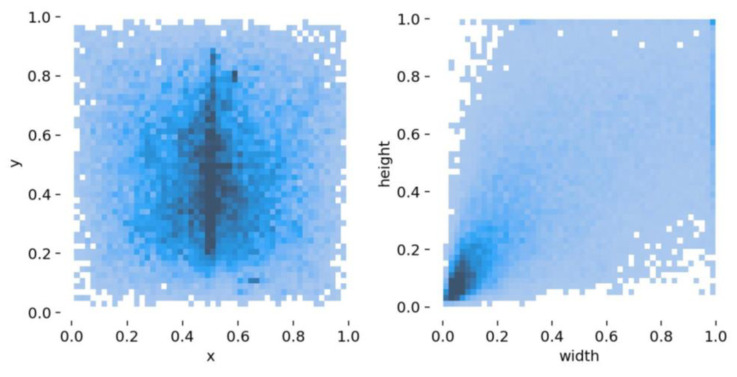
Bounding box distribution of the dataset. The darker the color, the greater the number.

Figure 3 presents the distribution of the bounding boxes in all images. We can see that a lot of bounding boxes are distributed in the center part of the image. From the distribution of height and width, we can find that most of the bounding boxes are small.

## 4. Proposed Detection Pipeline

DETR obtains comparable detection performance compared to the SOTA detection pipelines for the COCO dataset [52], in spite of a much briefer pipeline. However, the detection results for small objects are not as good as the big ones, due to the fact that the attention mechanism intends to model the overall information of the whole image, rather than local details. Figure 3 shows that a large number of defects account for less than a tenth of the entire image. This makes veneer defect detecting a challenging task for the original DETR. Furthermore, despite the fact that DETR needs much fewer manually designed components, such as anchors and non-maximum suppression, a new problem emerges, i.e., the format of positional encoding shall be predefined. However, the setting cannot be guaranteed to be the optimal one, since the selected format is subjective and depends on experience.

**Figure 4:**
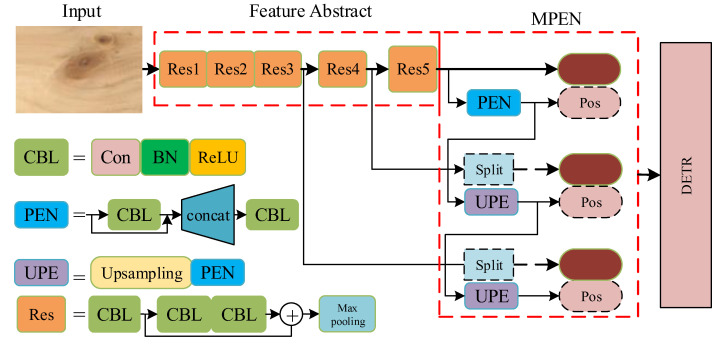
The architecture of the proposed detection pipeline.

According to the analyses above, this paper proposed a new detection pipeline based on DETR, namely the **m**ultiscale **p**osition **e**ncoding **n**et **d**etector (MPEND). The overall architecture is presented in Figure 4 (where ResX means the xth residual unit, Con mean the convolutional Layer, ReLU means rectified linear activation, BN means batch normalization and Pos means positional encoding). MPEND includes three parts, the feature abstraction backbone, multiscale position encoding net (MPEN) and a revised DETR detector. In the first step, following the original DETR, the feature abstraction module is constructed by the residual network. The input image is downsampled 32 times, and three different shapes of feature maps are obtained. In the second step, the obtained feature maps are integrated with positional encoding. Instead of designing positional encoding manually, MPEND utilizes a multiscale position encoding net to learn position information from the input image itself. In the last step, the feature map coupled with positional encoding are used as the input of the Transformer encoder. The detecting procedure is performed by the DETR with a modified loss function.

### 4.1. Backbone

The backbone of MPEND is constructed by the classical residual structure [53] with 15 convolutional layers, named RES15. A total of five residual structure are used, each of which have three convolutional layers followed by a max pooling layer. The main purpose of the max pooling layer is to downsample the input feature. In each convolutional layer, every convolutional unit is followed by the batch normalization. All the activation functions are ReLU. Only 1 × 1 and 3 × 3 convolutional kernels are adopted, following the experience of VGG. The details of the backbone are presented in Table 2.

Starting from the initial image $x_{\mathrm{img}}\in {\mathbb{R}}^{3\times H_{0}\times W_{0}}$ with three color channels, the backbone generates a lower-resolution activation map of $f\in {\mathbb{R}}^{C\times H\times W}$. Typically, for the basic model of MPEND with RES15, we have C=256 and $H,W=H_{0}/32,W_{0}/32$.

Many of the existing detection pipelines stack multiple residual units widely and deeply to improve the feature extraction capability of the backbone network. MPEND do not stack multiple residual units on the same layer to deepen the network width. This is because the feature extraction process of CNN-based pipelines almost entirely relies on the backbone network, while MPEND mainly obtains feature maps of different scales in the backbone network stage. Feature learning can also be realized in the following DETR model.

### 4.2. Multiscale Position Encoding Net

The original DETR used a feature map that is 32 times smaller than the input image. Although DETR can learn the overall relation of the whole map, the information of the small objects is dismissed. As a result, the small objects’ detection results of DETR are not as good as those of the big ones. Furthermore, both the original Transformer and the following DETR architecture adopted the trigonometric functions to generate, which is subjective and empirical. This section presents a multiscale position encoding net, including a multiscale feature maps module and an automatic position encoding net.

**Multiscale feature maps**. Multiscale feature maps have been verified to be an effective method for object detection. Similar to the tricks used in spatial PP and YOLOv3, three sizes of feature maps, $f_{1}\in {\mathbb{R}}^{C\times H_{1}\times W_{1}},f_{2}\in {\mathbb{R}}^{C\times H_{2}\times W_{2}},f_{3}\in {\mathbb{R}}^{C\times H_{3}\times W_{3}}$, from different layers of the backbone are adopted as the input of the following detection module. On the map with a smaller size, the detector can model the overall information to grasp the features of big objects, while on the map with a bigger size, the detector will focus on formulating the fine-grained features in the local parts, which is effective for small object detection. Typically, for an input image with 512 × 512 pixels, the three sizes of feature maps are 16 × 16, 32 × 32 and 64 × 64, with regard to $H_{1}\times W_{1},H_{2}\times W_{2}$ and $H_{3}\times W_{3}$, respectively.

One problem is that DETR only accepts input with a fixed length; hence, we collapse the spatial dimensions of $f_{1}$ into one dimension, resulting in a $C\times H_{1}W_{1}$ feature map.

**Figure 5:**
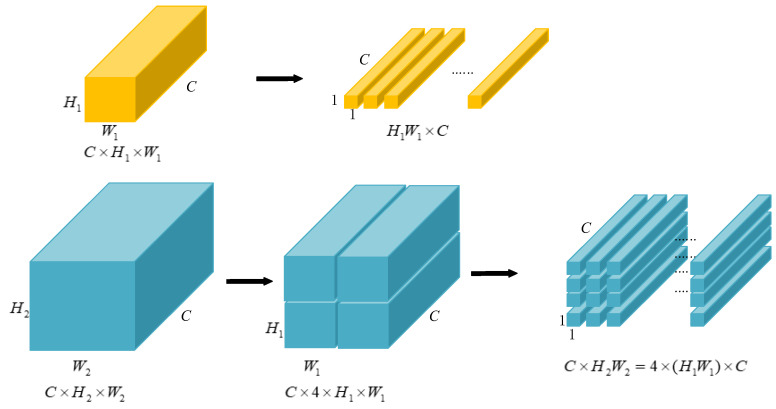
Feature map flattening and split.

In order to make the express style uniform and, furthermore, to facilitate the position encoding in the next step, the other two bigger maps are split into the size of $H_{1}\times W_{1}$. Figure 5 presents the flattening process for the smallest feature map, as well as the splitting results for the second feature map. The splitting process of the biggest feature map is similar to Figure 5. Typically, the feature map of $H_{3}\times W_{3}$ is first split into four $H_{2}\times W_{2}$ maps, then each of them is split the same as in Figure 5. The whole procedure is like a recursion.

It should be noted that the encoder and decoder used in the DETR detector are permutation-invariant. Therefore, from the feature view, split or not, the larger feature maps will not gain the amount of the feature information. However, this process is essential for the next position encoding.

**Position Encoding Net.** In the original Transformer, the sine and cosine functions are adopted for positional encoding. DETR adopts a generalization of the original Transformer encoding to the 2D case by independently using C/2 sine and cosine functions to yield a fixed absolute encoding to represent the spatial positions of images. Both of the encoding methods need manually designed formulas and introduce extra hyperparameters.

Given a picture of a bird, after a glimpse, we remember that bird and where it is. We have this memory not because we remember the position information, but because of the information it has in that area. That is, the information (or features) in different areas of an image itself has the position “encoding”. An intuitive assumption is that the information itself can be used to encode the position embedding.

**Figure 6:**
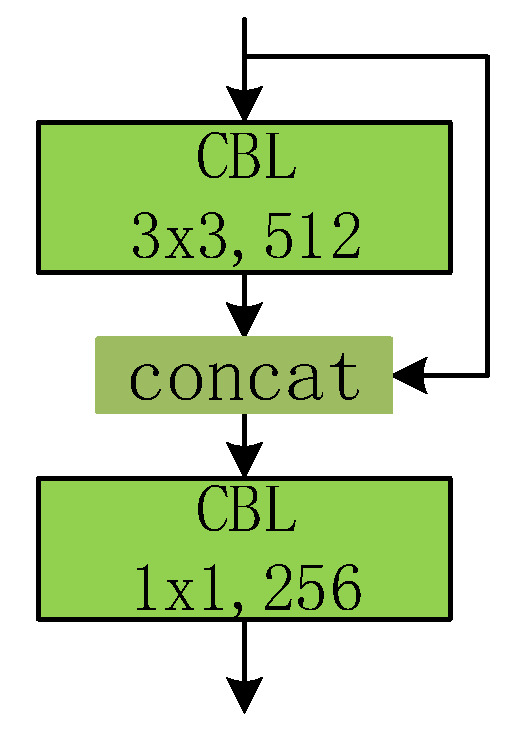
The positional encoding net.

Based on the analysis above, instead of directly encoding the location using extra formulas, we encode the position using information from different locations. Typically, for the feature map of the smallest size (16 × 16), a positional encoding branch is designed, as presented in Figure 6. The input of the positional encoding branch is the output feature map of the last residual block. After padding, the feature map passes through a convolution layer with 3 × 3 kernels. The resulting feature map is combined with the original input. The last convolution layer has 256 kernels with 1 × 1 size, in order to compress the dimension. The output size of the PEN is the same as the input feature map, which is essential for positional encoding.

**Figure 7:**
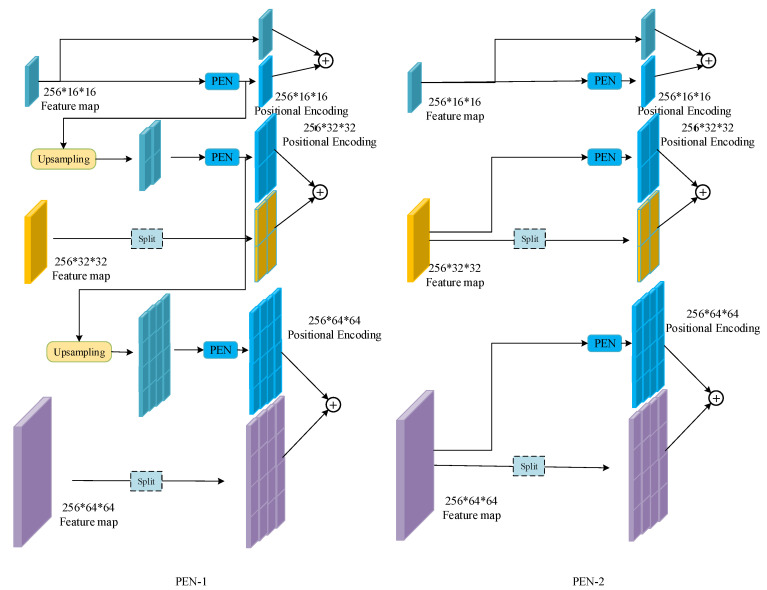
Two designs of the positional encoding net. * means multiple.

For the feature map with a larger size, two encoding strategies are designed, as presented in Figure 7. The first encoding strategy (named PEN-1) is presented in the architecture and is detailed on the left side of Figure 7. For the size $H_{1}\times W_{1}$, the feature map is directly inputted into the PEN for positional encoding. The result is marked as $Pe_{1}$. For the larger size $H_{2}\times W_{2}$, $Pe_{1}$ is first upsampled to be the same size as $H_{2}\times W_{2}$, then the upsampling result is inputted into PEN for positional encoding and the result is marked as $Pe_{2}$. Finally, $Pe_{2}$ is also upsampled to the size of $H_{3}\times W_{3}$, followed by the PEN. It should be noted that the three feature maps “approximately” share the same positional encoding map, since of all the input of the PEN is based on the smallest feature map. However, the three PENs used in the encoding processes are independent of each other.

The above description indicates that only the smallest feature map obtained from the last residual block is used for positional encoding. This is based on an intuitive assumption that the highly compressed feature is enough for the simple positional encoding. To verify this assumption, an extra encoding strategy (named PEN-2) is designed, as shown on the right side of Figure 7. For each feature map, the input of the PEN is the feature map obtained from the output of the corresponding residual block. The upsampling step is removed. The resulting positional encoding maps are independent of each other. The effectiveness of these two strategies will be compared in the next section.

### 4.3. Loss Functions

The original DETR includes four parts, the backbone based on the residual network, transformer encoder, transformer decoder and prediction feed-forward network. The definition of the loss function is one of the most import steps for object detection. DETR infers a fixed-size set of N predictions, then an optimal bipartite matching is conducted between the predicted and ground truth objects and, finally, the object-specific (bounding box) losses are optimized. The match cost between ground truth $y_{i}$ and prediction with index σ(i) is defined as:

(1) $$L_{match}(y_{i},{\overset{⌢}{y}}_{\sigma (i)})=-{Ι}_{\left\{c_{i}\neq \emptyset \right\}}{\overset{⌢}{p}}_{\sigma (i)}(c_{i})+{Ι}_{\left\{c_{i}\neq \emptyset \right\}}L_{box}(b_{i},{\overset{⌢}{b}}_{\sigma (i)})$$

where y is the ground truth set of objects and $\overset{⌢}{y}$ is the set of N predictions. ${\overset{⌢}{p}}_{\sigma (i)}(c_{i})$ is the probability of the prediction with index σ(i) of class $c_{i}$ and ${\overset{⌢}{b}}_{\sigma (i)}$ is the predicted box. Then, DETR finds a bipartite matching between the ground truth and the predictions by minimizing the following object function:

(2) $$\overset{⌢}{\sigma }=\arg\;\min\sum_{i}^{N}L_{match}(y_{i},{\overset{⌢}{y}}_{\sigma (i)})$$

After finding the optimal matching set, the total loss function is defined as:

(3) $$L(y,\overset{⌢}{y})=\sum_{i=1}^{N}\left[-\log{\overset{⌢}{p}}_{\overset{⌢}{\sigma }(i)}(c_{i})\right]+{Ι}_{\left\{c_{i}\neq \emptyset \right\}}L_{box}(b_{i},{\overset{⌢}{b}}_{\overset{⌢}{\sigma }}(i))$$

For the bounding box loss, a linear combination of the ${𝓁}_{1}$ loss and the generalized IoU loss [54] is adopted:

(4) $$L_{box}(b_{i},{\overset{⌢}{b}}_{\sigma (i)})=\lambda_{\mathrm{iou}}L_{\mathrm{iou}}(b_{i},{\overset{⌢}{b}}_{\sigma (i)})+\lambda_{{𝓁}_{1}}\left\|b_{i}-{\overset{⌢}{b}}_{\sigma (i)}\right\|$$

where $\lambda_{\mathrm{iou}},\lambda_{{𝓁}_{1}}\in \mathbb{R}$ are two hyperparameters.

The effectiveness of ${𝓁}_{1}$ loss has been proven in many machine learning problems. However, at the later stage of training, the loss function will fluctuate around the stable value, making it difficult to converge to a higher precision [53].

In object detection tasks, the intersection ratio IoU is one of the most used metrics for performance evaluation. A higher value of IoU means a more accurate prediction result of the model. In the training stage, IoU can be used as the basis for dividing positive and negative samples in the anchor-based method. It can also be used in the loss function. However, IoU has a serious defect: if there is no overlap between two targets, IoU will be 0 and will not calculate the distance between two targets. In the case of such non-overlapping targets, if IoU is used as a loss function, the gradient will be 0, which cannot be optimized.

To overcome these drawbacks, Complete-IOU (CIoU) [54] was proposed. In CIoU, a term was added to the end of IoU to calculate the minimum external rectangle of the two boxes, which is used to calculate the distance between the two boxes. This solves the problem of zero gradient when the two objects do not intersect. Furthermore, the standardized distance of the center points of the two Bboxes is minimized to accelerate the convergence process. At the same time, the aspect ratio of the boxes was also introduced to further measure the shape of the boxes. CIoU has been verified to achieve better convergence speed and accuracy for bounding box prediction problems. Here, we redefine the bounding box loss of DETR as

(5) $$L_{box}(b_{i},{\overset{⌢}{b}}_{\sigma (i)})=1-(IoU-\frac{\rho^{2}(b_{i},{\overset{⌢}{b}}_{\sigma (i)})}{\gamma^{2}}-\frac{\nu^{2}}{(1-IoU)+v})$$

where ρ is the Euclidean distance between the center of the ground truth and the prediction. γ represents the diagonal distance of the smallest enclosing rectangle. ν is a penalty term considering the ratio of width and the height, i.e.,

(6) $$v=\frac{4}{\pi^{2}}{\left(\arctan\frac{w_{i}}{h_{i}}-\arctan\frac{w_{\sigma (i)}}{h_{\sigma (i)}}\right)}^{2}$$

From Equation (5), we can find that the two extra hyperparameters are removed and the loss function comprehensively considers the shape of the ground truth and the predictions.

## 5. Experiments and Analyses

In this section, the effectiveness of the proposed method is validated. The experiments mainly consist of three parts. First, the proposed multiscale positional encoding net detector (MPEND) is compared with some of the related state-of-the-art object detection pipelines. Then, some ablation studies are carried out to compare the performance of the proposed learning skills.

### 5.1. Experimental Settings

**Experiment environment.** The deep learning framework used in this paper is Pytorch. The integrated development environment is Pycharm with a version of 11.0.4. The platform has an Intel Core i7-9750 @ 2.60 GHz CPU, 32 Gb RAM, Nvidia Quadro RTX5000 GPU. 

**Parameter setting.** Three of the state-of-the-art object detection pipelines are adopted for performance comparison, i.e., DETR, Faster-RCNN and YOLOv4. For Faster-RCNN and YOLOv4, the hyperparameters are set as in the original papers. ResNet-50 is used as backbone for DETR and Faster-RCNN. For MPEND, except for the base backbone described in Section 4.1, ResNet-50 is also used for deep comparison with DETR; the corresponding model is called MPEND-R50. The number of object queries of DETR and MPEND is set to be 20, rather than 100 as in the original paper, since the defect number on each veneer image is much smaller than the COCO dataset. For MPEND, if not specified, the PEN adopted is PEN-1. Both the initial learning rate and weight decay are 10^−4^. The existing works presented compelling suggestions for the hyperparameters for Transformer. The proposed MPEND is derived from the other MPEND, so the other hyperparameters of MPEND are the same as DETR. Specially, both the encoder and decoder number are set to be 6, the learning rate drops after 40 epochs and the classification cost is 2. According to Equation (5), the two hyperparameters (i.e., $\lambda_{\mathrm{iou}},\lambda_{{𝓁}_{1}}$) are removed. All the 4 pipelines are trained on the dataset described in Section 3 with 200 epochs with a batch size of 8.

**Evaluation metrics.** The most commonly used average precision (AP) and mean average precision (mAP) are used as evaluation metrics. Let IoU refer to the ratio of the intersection between the prediction box and the real box and their union. When the value of IoU is greater than the threshold we set, the prediction box is considered correct; otherwise, the prediction is wrong. Let TP be the number of positive samples that are correctly detected, FP be the number of negative samples that are incorrectly detected and FN be the number of positive samples that are incorrectly detected. Then, AP and mAP can be formulated as:

(7) $$\begin{array}{l} p=\frac{Tp}{Tp+Fp},r=\frac{Tp}{Tp+Fn}\\ p_{\mathrm{interp}}(r_{i+1})=\underset{r^{\prime }:r^{\prime }\geq r_{i+1}}{\max}p(r^{\prime })\\ AP=\int_{0}^{1}p(r)dr\approx \sum_{i}\left(r_{i+1}-r_{i}\right)p_{\mathrm{interp}}(r_{i+1})\\ mAP=\frac{1}{n}\sum_{i=1}^{n}AP_{i} \end{array}$$

where p donates precision and r denotes recall, while n is the number of categories.

Following the standard criterion of the COCO dataset, three thresholds are selected, resulting in 3 metrics, mAP50, mAP75 and mAP50:5:95. The thresholds adopted for the 3 metrics are 0.5, 0.75 and from 0.5 to 0.95 with a step of 0.05, respectively. The details can refer to the COCO dataset. Using 3 different thresholds can show the results for different scales of defects more clearly. Moreover, the confusion matrix is also used to analyze the performance for different categories.

### 5.2. Performance Comparison

The detection results for the veneer defect dataset described in Section 3 are presented in Table 3. With regard to the results for every detect class, MPEND-R50 (MPEND with ResNet-50) obtains the best result for the live knot defect. For the other two defect classes, MPEND-R50 also obtains adequate performances compared to the other two SOTA methos, Faster-RCNN and DETR. For two defect classes, live knot and dead knot, MPEND-R50 has better performances than DETR, though for the wormhole class, MPEND-R50 has only a 0.3% decrease compared to DETR. With regard to the overall metrics, it is not hard to see that MPEND-R50 wins two out of three entries. Especially for the mAP50:5:95 metric, MPEND-R50 obtains a 1.7% improvement compared to the sub-optimal method. Even for the mAP75, MPEND-R50 is comparable to the best performance. As a whole, MPEND (backbone is RES15) exhibits the worst performance, which may be due to the simple feature abstraction backbone. However, the accuracy of MPEND is adequate for realistic application.

**Figure 8:**
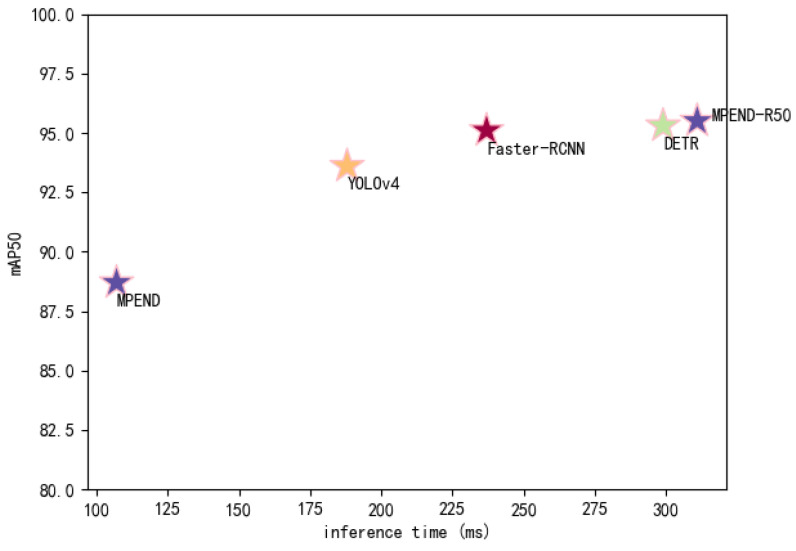
Comparison of inference speed and detection accuracy.

A more comprehensive comparison of the accuracy and detection speed of the five models is presented in Figure 8. The result shows that MPEND-R50 obtains the best detection accuracy, while the inference time is slightly longer compared to DETR. However, the inference time of MPEND-R50 is much longer than the other methods. Although MPEND gives the worst accuracy, the inference time is nearly three times faster than DETR and MPEND-R50. This excellent inference time coupled with adequate detection accuracy make MPEND a promising method for engineering application.

**Figure 9:**
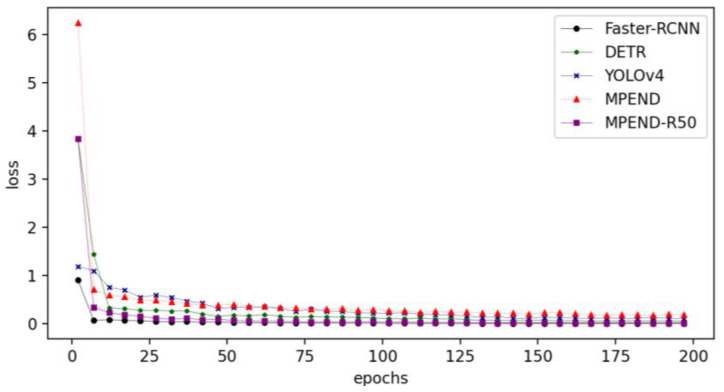
Loss curves of 5 pipelines.

The loss curves of the five detectors are presented in Figure 9. It is not hard to see that Faster-RCNN has the fastest convergency speed. The MPEND-R50 has the second-fastest convergency speed, followed by DETR. This indicates that the tricks adopted by the MPEDN-R50 are helpful for training. Although the convergency speed of MPEND is much faster than YOLOv4 at the beginning stage, the loss curve is premature convergence, which indicates that it falls into local optimal. The difference between the two proposed models also indicates that a strong and deep backbone is essential for the detector.

In conclusion, with an adequate detection accuracy, MPEND is much faster than the state-of-the-art detectors. On the other hand, combined with a strong feature extraction backbone, MPEND-R50 presents much better performance with almost negligible extra time consumption. All these indicate that the proposed tricks are effective for defect detection problems.

### 5.3. Ablation Experiments

In this part, three ablation experiments are conducted to further analyze the effectiveness of the tricks adopted in MPEND and MPEND-R50. It should be noted that all the adopted models in this part are MPEND in order to save on calculation consumption.

**Position encoding.** In order to verify the assumption in Section 4.2, the performances of the two position encoding strategies are compared. The backbone is R15 and the other settings are same as for MPEND, except for the position encoding part. The results are presented in Table 4. From Table 4, we can see that the PEN-2 encoding trick causes a substantial decrease in the performance of the detector compared to PEN-1. This indicates that the fine-gained features are not useful for position encoding. In PEN-2, the input of every PEN is the corresponding feature map. During training, the fine-gained feature “confuses” the encoding net so that PEN cannot abstract the position information. In fact, PEN degenerates into a feature extraction network rather than one for position encoding.

**Figure 10:**
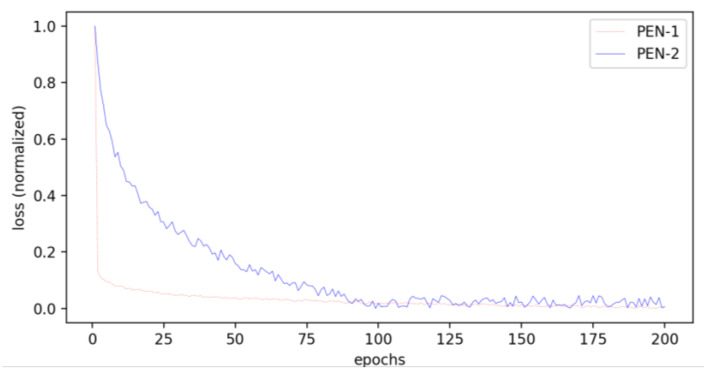
Loss curves of PEN-1 and PEN-2.

Figure 10 presents the loss curves of PEN-1 and PEN-2. The losses of the two models are normalized for apparent comparison. We can see that the convergency speed of PEN-2 is much slower than that of PEN-1. Furthermore, the oscillation of the loss curve also indicates that PEN-2 is unable to learn a serviceable position encoding.

**Multiscale feature map.** The effectiveness of the multiscale feature map is verified. In order to reduce the influence of position encoding, three models are designed for comparison, named MPEND-S, MPEND-M and MPEND-L, with a feature map of 16 × 16, 32 × 32 and 64 × 64, respectively. For all of the three models, only the 16 × 16 feature map is used for position encoding, following the conclusion obtained in the above ablation experiment. The results are presented in Table 5.

We can see that MPEND with three feature maps outperforms all of the models with a single feature map. This indicates that a multiscale feature map is an essential trick for multiscale object detection. Furthermore, Table 3 also shows that the bigger the feature map, the better the performance. There are two reasons. The first is that a small feature confuses the network to learn position encoding, as explained in the above ablation experiment. The second is that a larger feature map can supply more fine-gained information.

**Figure 11:**
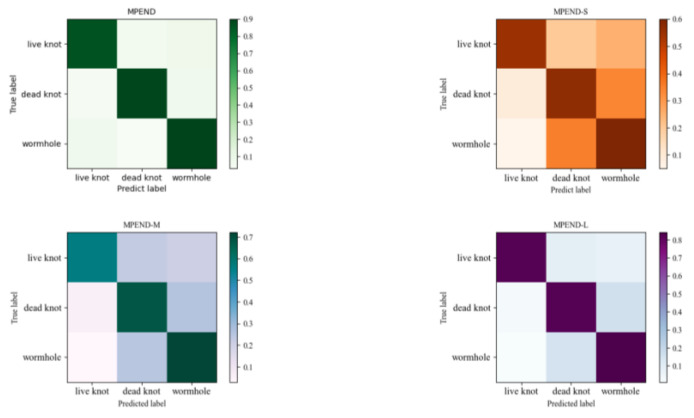
Confusion matrix of models with different feature maps.

The confusion matrix results in Figure 11 also show that MPEND obtains the best performance compared to other single feature map-based models. Furthermore, we can also see that the false positive samples of live knots come from both the other two categories, while the false positive samples of dead knots and wormholes mainly come from each other.

**Figure 12:**
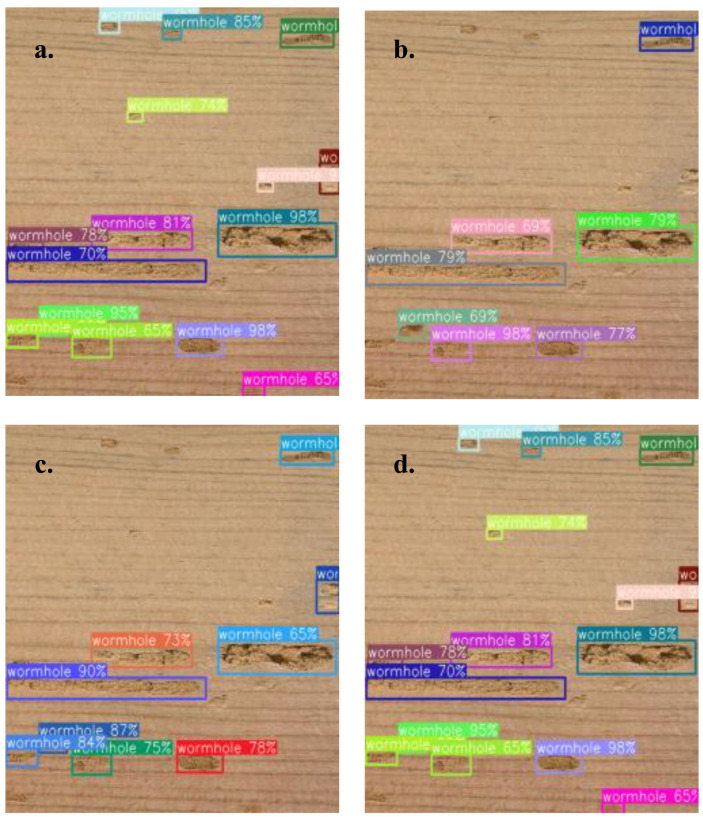
An example of detection results of four different models. (**a**) MPEND, (**b**) MPEND-S, (**c**) MPEND-M, (**d**) MPEND-L.

Figure 12 presents an example of detection results for a veneer full of different size of wormholes. It can be seen that MPEND detects most of the defects. MPEND-S loses many of the small defects. With the increase in the feature map, an apparent performance improvement is presented. All these results indicate that multiscale is essential for the detection of objects with different shapes.

**Loss function.** For MPEND, we keep the other parts invariant but replace the loss function with the original one used in DETR. The new model is denoted as MPEND-L1. The results are presented in Table 6. We can see that the detector with the new designed loss function performs a little better than the one using the original loss function. This indicates that the new loss function contributes slightly to the performance. This also indicates that the other two tricks, i.e., multiscale feature map and position encoding net, contribute to the performance gain in a substantial way.

## 6. Conclusions

Wood is one of the main building materials. While wood resources are depletable, defects on veneers result in substantial waste. Existing veneer defect detection relies on manual experience or photoelectric-based methods, which are either subjective and inefficient or need a lot of investment. Computer vision-based object detection methods have been used in many realistic areas. One of the state-of-the-art detectors, DETR, shows amazing performance in many applications. However, the position encoding formulas need to be manually designed. Furthermore, DETR fails to detect small objects. Based on these analyses, this paper proposes a new deep learning defect detection pipeline. First, an image collection device is constructed and a total of more than 16,380 defect images are collected through a mixed data augmentation method. In the feature extraction stage, multiscale feature maps are used for detecting objects with different sizes. A position encoding net is designed to replace the original manually designed methods. The loss function is also redefined for much more stable training. From the speed perspective, the accuracy of MPEND is 6% lower than the best model, but it is more than two times faster. From the accuracy perspective, MPEND-R50 is an improvement of 1.4% compared to the best model, with a similar detection speed. The results indicate that the proposed multiscale feature maps and positional encoding strategy are effective for detection. Without designing positional encoders manually, more integrated approaches can be explored.

Even though the detection results of the proposed method are adequate compared to SOTA, the detection speed is a bottleneck for realistic application. The experiments also show that MPEND does not balance the detection accuracy and the speed. Future work will focus on improving the detection speed even on larger images and trying other, much more effective, backbones. Secondly, the results indicate the effectiveness of the positional encoding net, but we cannot prove this is the best strategy in a mathematical way. There are many more suitable positional encoding strategies that still need to be explored. Furthermore, the explored defect methods of this manuscript are all on the surface. Traditional methods, such as X-ray, can directly look at the inside of wood. How to combine these two kinds of methods is also an interesting problem.

## Figures and Tables

**Table 1:** Dataset description.

Defect	No. of Training Image/Label	No. of Validation Image/Label	No. of Test Image/Label	Total Image/Label
Live knot	4200/4830	600/690	1200/1380	6000/6900
Dead knot	3612/4045	516/577	1032/1155	5160/5777
Wormhole	3654/4498	522/642	1044/1285	5220/6425

**Table 2:** Details of the backbone of the basic model.

Unit	Type	No. of Conv	Size/Step	Output Size
Res1	Con	512	3 × 3/1	256 × 256
	Con	256	1 × 1/1	
	Con	512	3 × 3/1	
	Pooling	-	2 × 2/2	
Res2	Con	512	3 × 3/1	128 × 128
	Con	256	1 × 1/1	
	Con	512	3 × 3/1	
	Pooling	-	2 × 2/2	
Res3	Con	256	3 × 3/1	64 × 64
	Con	128	1 × 1/1	
	Con	256	3 × 3/1	
	Pooling	-	2 × 2/2	
Res4	Con	512	3 × 3/1	32 × 32
	Con	256	1 × 1/1	
	Con	512	3 × 3/1	
	Pooling	-	2 × 2/2	
Res5	Con	256	3 × 3/1	16 × 16
	Con	128	1 × 1/1	
	Con	256	3 × 3/1	
	Pooling	-	2 × 2/2	

**Table 3:** Detection results of the proposed methods compared with 3 state-of-the-art detection pipelines.

Model	AP50			mAP50	mAP75	mAP50:5:95
	Live Knot	Dead Knot	Wormhole			
Faster-RCNN	93.4	95.2	96.7	95.1	72.6	60.2
YOLOv4	91.6	93.7	95.4	93.6	68.0	58.4
DETR	94.1	94.5	97.2	95.3	73.1	60.4
MPEND	86.9	89.1	90.2	88.7	59.6	43.8
MPEND-R50	94.7	95.0	96.9	95.5	71.2	62.1

**Table 4:** Detection results of the two position encoding strategies.

Model	AP50			mAP50	mAP75	mAP50:5:95
	Live Knot	Dead Knot	Wormhole			
PEN-1	86.9	89.1	90.2	88.7	59.6	43.8
PEN-2	63.4	65.8	73.1	67.4	32.9	20.2

**Table 5:** Detection results of detectors with different feature map sizes.

Model	AP50			mAP50	mAP75	mAP50:5:95
	Live Knot	Dead Knot	Wormhole			
MPEND	86.9	89.1	90.2	88.7	59.6	43.8
MPEND-S	55.3	57.7	60.6	57.8	26.1	17.4
MPEND-M	62.6	68.5	72.4	67.8	33.4	19.3
MPEND-L	82.0	81.9	84.7	82.7	36.8	23.8

**Table 6:** Detection results of detector with different loss functions.

Model	AP50			mAP50	mAP75	mAP50:5:95
	Live Knot	Dead Knot	Wormhole			
MPEND	86.9	89.1	90.2	88.7	59.6	43.8
MPEND-L1	87.0	88.7	88.9	88.2	57.2	43.1

## Data Availability

The datasets of the current study are available from the corresponding author upon reasonable request.

## Nomenclature

The following nomenclature is used in this manuscript:

DETR	DEtection TRansformer
IoU	Intersection over union
CNN	Convolutional neural networks
ILSVRC	ImageNet large-scale visual recognition challenge
SOTA	State-of-the-art
RCNN	Region-CNN
YOLO	You only look once
RNN	Recurrent neural network
ViT	Vision transformer
MPEND	Multiscale position encoding net detector
MPEN	Multiscale position encoding net
RES	Residual net
PEN	Position encoding net
